# Ethanol Extract of *Rosa rugosa* Ameliorates Acetaminophen-Induced Liver Injury via Upregulating Sirt1 and Subsequent Potentiation of LKB1/AMPK/Nrf2 Cascade in Hepatocytes

**DOI:** 10.3390/molecules28217307

**Published:** 2023-10-28

**Authors:** Yecheng Lei, Xiao Lei, Anqi Zhu, Shijie Xie, Tiantian Zhang, Chuo Wang, Anning Song, Xiaoming Wang, Guangwen Shu, Xukun Deng

**Affiliations:** 1 School of Pharmaceutical Sciences, South-Central Minzu University, Wuhan 430074, China; rayyee5960@gmail.com (Y.L.);; 2 School of Life Sciences, Nanjing University, Nanjing 210023, China; wangxm07@nju.edu.cn

**Keywords:** extract of *Rosa rugosa*, acetaminophen, liver injury, Sirt1, Nrf2

## Abstract

Acetaminophen (APAP)-induced liver injury is a common hepatic disease resulting from drug abuse. Few targeted treatments are available clinically nowadays. The flower bud of *Rosa rugosa* has a wide range of biological activities. However, it is unclear whether it alleviates liver injury caused by APAP. Here, we prepared an ethanol extract of *Rosa rugosa* (ERS) and analyzed its chemical profile. Furthermore, we revealed that ERS significantly ameliorated APAP-induced apoptosis and ferroptosis in AML-12 hepatocytes and dampened APAP-mediated cytotoxicity. In AML-12 cells, ERS elevated Sirt1 expression, boosted the LKB1/AMPK/Nrf2 axis, and thereby crippled APAP-induced intracellular oxidative stress. Both EX527 and NAM, which are chemically unrelated inhibitors of Sirt1, blocked ERS-induced activation of LKB1/AMPK/Nrf2 signaling. The protection of ERS against APAP-triggered toxicity in AML-12 cells was subsequently abolished. As expression of LKB1 was knocked down, ERS still upregulated Sirt1 but failed to activate AMPK/Nrf2 cascade or suppress cytotoxicity provoked by APAP. Results of in vivo experiments showed that ERS attenuated APAP-caused hepatocyte apoptosis and ferroptosis and improved liver injury and inflammation. Consistently, ERS boosted Sirt1 expression, increased phosphorylations of LKB1 and AMPK, and promoted Nrf2 nuclear translocation in the livers of APAP-intoxicated mice. Hepatic transcriptions of HO-1 and GCLC, which are downstream antioxidant genes of Nrf2, were also significantly increased in response to ERS. Our results collectively indicated that ERS effectively attenuates APAP-induced liver injury by activating LKB1/AMPK/Nrf2 cascade. Upregulated expression of Sirt1 plays a crucial role in ERS-mediated activation of LKB1.

## 1. Introduction

Acetaminophen (APAP) is a widely used non-steroidal anti-inflammatory drug worldwide [1]. Overdose of APAP results in the accumulation of its toxic metabolites in the liver, leading to intrahepatic oxidative stress, massive cell death, intense inflammatory reactions, and ultimately severe liver damage [2]. The incidence of APAP-triggered liver injury, which has become a common clinical liver disease caused by drug abuse, has elevated recently [3]. Without timely intervention, APAP-provoked liver damage may deteriorate into fatal liver failure [2]. At present, N-acetyl cysteine (NAC) is mainly used to treat APAP-provoked liver injury clinically. However, its therapeutic effects are generally not satisfactory [4,5,6]. Therefore, it is still necessary to search for new bioactive substances with the capacity to alleviate liver injury triggered by APAP.

Apoptosis is an important form of cell death [7] that plays a crucial role in APAP-provoked massive cell death in the liver [8]. In hepatocytes, APAP increases expressions of proapoptotic factor Bax and downregulates those of antiapoptotic factor Bcl-27. These events further activate caspase cascades, including caspase-3, -8, and -9, ultimately leading to cell apoptosis [9]. It has been reported that knockout of Bax or overexpression of Bcl-2 exerts inhibitory effects on APAP-induced liver injury [7,10]. Ferroptosis is a different modality of cell death from apoptosis, characterized by iron-dependent lipid peroxidation. During ferroptosis, levels of lipid peroxidation products, such as 4-HEN, MDA, and LPO, are increased [11,12,13]. Recent studies unveiled close connections between hepatocyte ferroptosis and APAP-provoked liver injury [14,15,16]. Suppressing the ferroptosis of hepatocytes is implicated in the hepatoprotective activities of some natural products against APAP [17,18,19].

Nrf2 is a transcription factor exerting positive regulatory effects on cellular antioxidant capacity and has been widely identified as a therapeutic target for diseases related to oxidative stress [20,21]. In a resting state, Nrf2 binds to the Keap1 protein and localizes in the cytoplasmic matrix [21]. Intracellular oxidative stress results in conformational changes of Keap1 and subsequent disassociation of Nrf2 from Keap1. Then, Nrf2 translocates into nuclei and promotes transcriptions of its responsive genes such as HO-1 and GCLC. These factors are beneficial for clearing intracellular reactive oxygen species (ROS) and elevating antioxidative capacity [22]. Previous research unveiled that the sensitivity of animals to APAP is negatively correlated with Nrf2 activity in the liver [16,23]. Similarly, hepatic overexpression of Nrf2 effectively alleviates APAP-induced liver injury [24], and some antioxidants, such as urolithin A, that are capable of increasing Nrf2 activity decline the hepatotoxicity of APAP [25]. The findings above suggest that the potentiation of Nrf2 can be a promising approach for intervening in APAP-triggered liver injury.

Nrf2 cascade is regulated by upstream signals such as LKB1/AMPK [26]. Activation of AMPK promotes nuclear translocation of Nrf2 and potentiates Nrf2 [27]. Inhibiting the catalytic activity of AMPK blocks Nrf2 potentiation mediated by some bioactive substances [28]. LKB1 catalyzes AMPK phosphorylation and activates AMPK, thereby inducing Nrf2 translocation into nuclei and initiating transcriptions of its downstream genes [26]. Sirt1 is a protein deacetylase that promotes mitochondrial biogenesis and ATP synthesis by catalyzing the deacetylation of some of its target proteins. In most cells, Sirt1 has the function of promoting cell proliferation, antagonizing oxidative stress, and inhibiting cell death. APAP intoxication is accompanied with decreased Sirt1 in the liver, and the activation of Sirt1 is conducive to the amelioration of APAP-induced liver injury [29,30]. Mechanistically, Sirt1 plays a crucial role in regulating the activity of LKB1/AMPK [26,31]. It is widely accepted that Sirt1 promotes LKB1 phosphorylation, which activates LKB1 and its downstream events [31]. Knockout of Sirt1 cripples phosphorylations of LKB1 and AMPK [32]. In myocardial cells, enhancing Sirt1/LKB1 interaction activates the LKB1/AMPK axis [31]. In mast cells, the knockdown of Sirt1 eliminates LKB1/AMPK activation triggered by tanshinone IIA [33]. In the liver, a decrease of Sirt1 inhibits Nrf2 activity, exacerbating APAP-induced oxidative stress and liver injury [34,35]. The aforementioned observations indicate that Sirt1 has the potential to activate Nrf2 via LKB1/AMPK, thereby exerting hepatoprotective effects against APAP.

Rose (*Rosa rugosa*) constitutes a commonly used herbal medicine with a wide range of biological activities, especially in its dried flower buds. It boosts expressions of antioxidant enzymes and inhibits lipid peroxidation in livers [36]. It also improves hepatic lipid metabolism via modulating AMPK [37]. However, it is currently unclear whether rose has the capacity to alleviate APAP-induced liver injury and modulate hepatic Nrf2 signaling. Thus, we prepared an ethanol extract of *Rosa rugosa* (ERS), analyzed its characteristic chemical components, evaluated the protective effects of ERS on APAP-induced liver injury in mice, and explored the underlying molecular mechanisms.

## 2. Results

### 2.1. The Chemical Profile of ERS

**Figure 1:**
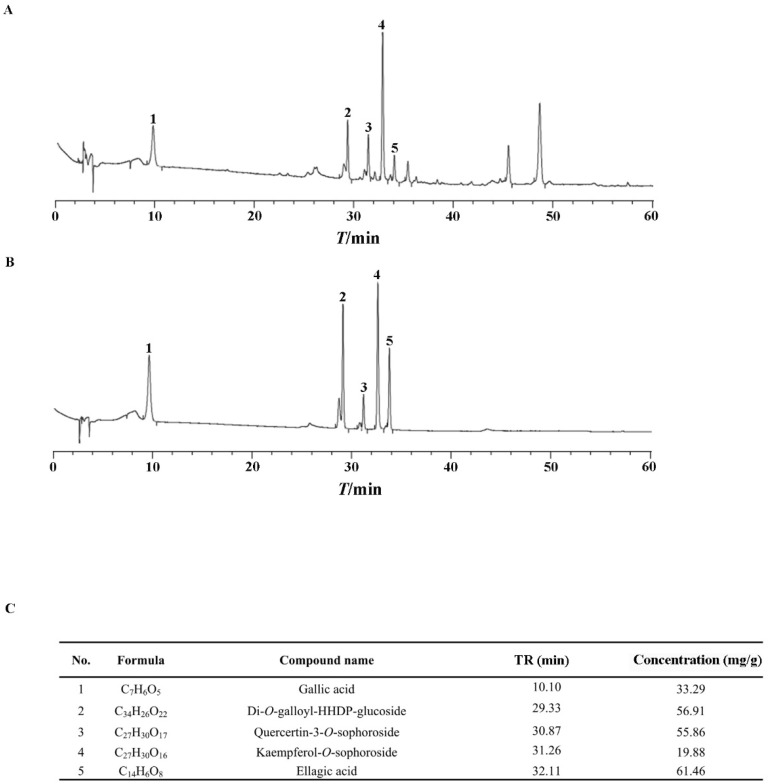
Chemical composition analysis of ERS. (**A**,**B**) ERS (**A**) and mixed standards of characteristic compounds in ERS (**B**) were analyzed by HPLC. (**C**) Contents of characteristic polyphenols in ERS were determined by HPLC.

As clarified previously, polyphenols are the active ingredients in ERS [37,38,39]. We showed that the total polyphenol content in ERS was 47.3%. Five active polyphenols, including gallic acid, Di-O-galloyl-HHDP-glucoside, quercetin-3-*O*-sophoroside, kaempferol-3-*O*-sophoroside, and ellagic acid, were identified in ERS and analyzed quantitatively using mixed standards of these compounds as references (Figure 1). Data of our chemical composition exploration are comparable to those of previous reports [40,41,42], indicating the reliability of the process to prepare and analyze ERS.

### 2.2. ERS Ameliorates APAP-Provoked Apoptosis and Ferroptosis in AML-12 Hepatocytes

**Figure 2:**
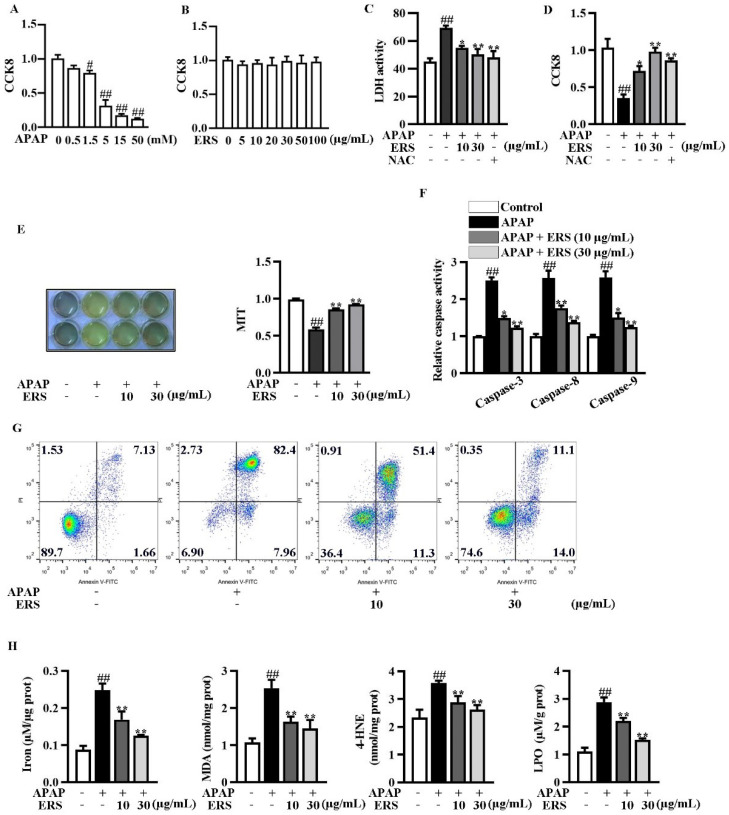
ERS ameliorated APAP-provoked apoptosis and ferroptosis in AML-12 hepatocytes. (**A**,**B**) AML-12 hepatocytes were treated with the indicated concentrations of APAP (**A**) or ERS (**B**) for 24 h, and then subjected to CCK8 assay. (**C**,**D**) AML-12 hepatocytes were incubated with ERS (10 and 30 μg/mL) or NAC (10 mM) for 24 h. Then, cells were treated with 5 mM APAP for another 24 h. Finally, cytotoxicity was determined by LDH (**C**) and CCK8 (**D**) assay. (**E**–**H**) AML-12 cells were incubated with ERS (10 and 30 μg/mL) for 24 h and treated with 5 mM APAP for another 24 h. Then, cell viability was determined by MTT assay (**E**). Both MTT staining results (left) and statistical results (right) were shown. Cell apoptosis was detected by caspase activity assay (**F**) and FACS (**G**). Cell ferroptosis was detected by measuring related parameters such as iron, MDA, 4-HEN, and LPO (**H**). ^#^ *p* < 0.05, ^##^ *p* < 0.01, compared to cells treated by vehicle. * *p* < 0.05, ** *p* < 0.01, compared to cells treated by APAP alone.

We evaluated the cytotoxicity of the APAP and ERS on AML-12 hepatocytes using CCK8 assay. The cytotoxicity of APAP elevated with the increase of its concentration (Figure 2A), whereas the cytotoxicity of ERS was undetectable (Figure 2B). As shown by LDH and CCK8 assays, ERS considerably reduced APAP-provoked cytotoxicity in AML-12 hepatocytes, and the ameliorative effects of ERS were comparable to those of NAC (Figure 2C,D). ERS-mediated protection against APAP was further confirmed by MTT assay (Figure 2E). APAP stimulation led to apoptosis of AML-12 cells, which was dramatically relieved by ERS (Figure 2F,G). The detection of intracellular iron, MDA, 4-HNE, and LPO unveiled that ferroptosis occurred in response to APAP. ERS reversed APAP-induced ferroptosis in AML-12 hepatocytes (Figure 2H). The findings above indicated that ERS can dampen APAP-mediated cytotoxicity in AML-12 hepatocytes.

### 2.3. ERS Elevates Sirt1 Expression and Activates LKB1/AMPK/Nrf2 Axis in AML-12 Cells

**Figure 3:**
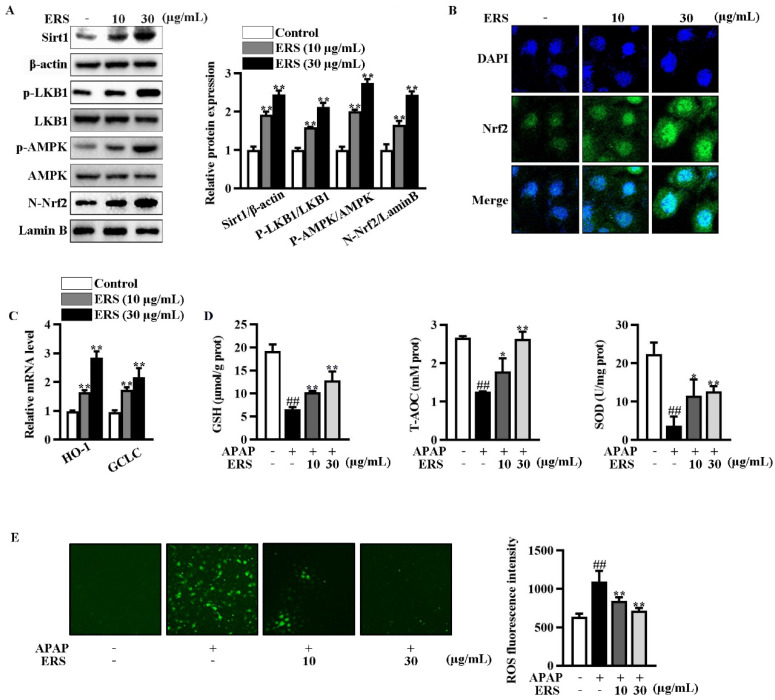
ERS elevated Sirt1 expression and activated the LKB1/AMPK/Nrf2 axis in AML-12 cells. (**A**–**C**) AML-12 hepatocytes were incubated with the indicated concentrations of ERS for 24 h. Then, protein levels of intracellular Sirt1, phosphorylation of LKB1 and AMPK, and Nrf2 in nuclei were detected by immunoblotting (**A**). Both immunoblotting images (**left**) and statistical results (**right**) were shown. Nuclear accumulation of Nrf2 was also detected by confocal microscope. (**B**). Transcriptions of HO-1 and GCLC were detected by real-time PCR (**C**). (**D**,**E**) AML-12 cells were incubated with ERS (10 and 30 μg/mL) for 24 h, and challenged with APAP (5 mM) for another 24 h. Then, intracellular antioxidative parameters including GSH, T-AOC, and SOD were determined (**D**). ROS were determined by fluorescence staining (**E**, left) and fluorescence spectrophotometry assay (**E**, right). ^##^: *p* < 0.01, compared to cells treated by vehicle (**D**,**E**). * *p* < 0.05, ** *p* < 0.01, compared to cells treated by vehicle (**A**,**C**) or APAP alone (**D**,**E**).

Immunoblotting showed that ERS increased Sirt1 expression and phosphorylation of LKB1 and AMPK in AML-12 cells (Figure 3A). ERS also promoted nuclear translocation of Nrf2 (Figure 3A,B). Transcriptions of typical Nrf2-responsive genes, including HO-1 and GCLC, were thereby boosted (Figure 3C). Consistently, ERS crippled reductions of intracellular antioxidant capacity and elevations of ROS resulting from APAP intoxication (Figure 3D,E). Collectively, our data indicated that ERS elevates Sirt1 expression and potentiates LKB1/AMPK/Nrf2 antioxidant signing in AML-12 hepatocytes.

### 2.4. Inhibition of Sirt1 Eliminates ERS-Mediated Potentiation of LKB1/AMPK/Nrf2 Axis

**Figure 4:**
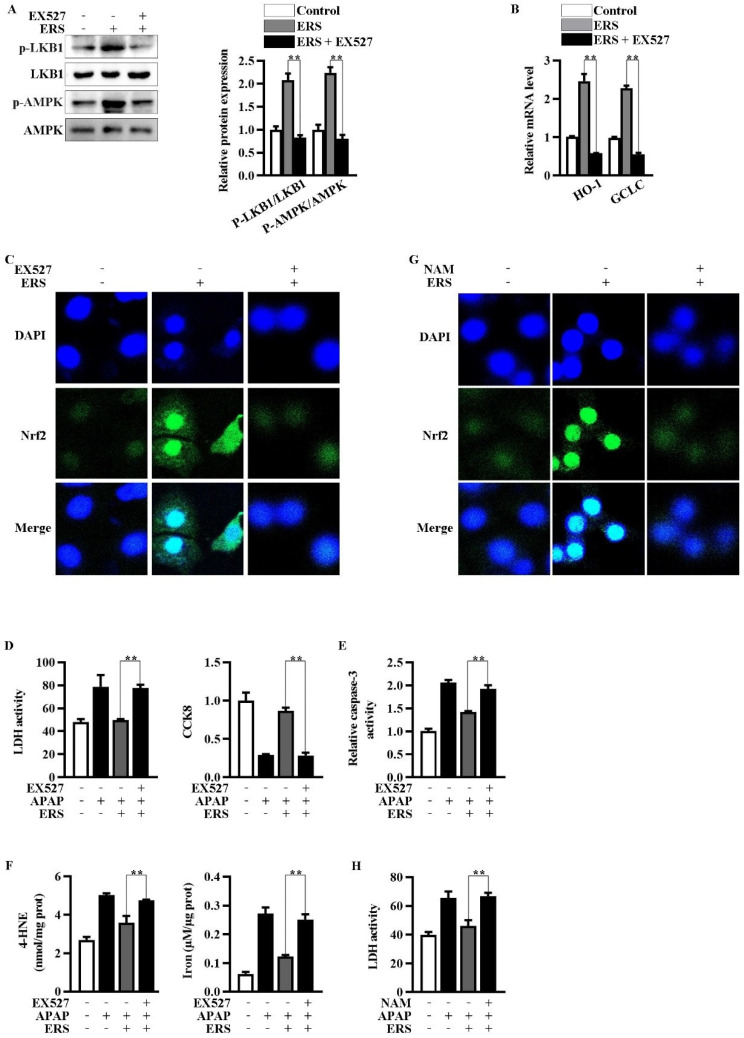
Inhibition of Sirt1 eliminated ERS-mediated potentiation of LKB1/AMPK/Nrf2 axis. (**A**–**C**) AML-12 cells were subjected to the indicated treatments. Then, phosphorylation of LKB1 and AMPK was measured by immunoblotting (**A**). Transcriptions of HO-1 and GCLC were detected by real-time PCR (**B**). Nuclear translocation of Nrf2 was detected by confocal microscope (**C**). (**D**–**F**) Cells were subjected to the indicated treatments. Then, cytotoxicity (**D**), apoptosis (**E**), and ferroptosis (**F**) were quantified. (**G**,**H**) AML-12 hepatocytes were subjected to the indicated treatments. Levels of Nrf2 in nuclei were determined by confocal microscope (**G**). Cytotoxicity was quantified by LDH assay (**H**). ** *p* < 0.01, compared with the indicated control (cells treated with ERS and APAP, but no Sirt1 inhibitor).

Inhibition of Sirt1 by EX527 resulted in an abrogation of ERS-mediated activation of LKB1/AMPK (Figure 4A). Upregulations of Nrf2 in nuclei and transcription of HO-1 and GCLC were, in turn, eliminated by EX527 (Figure 4B,C). NAM is another Sirt1 inhibitor whose chemical structure is unrelated to that of EX527. NAM also antagonized the nuclear translocation of Nrf2 induced by ERS (Figure 4G). Additionally, both EX527 and NAM reversed the protective effects of ERS on APAP-provoked cytotoxicity in AML-12 hepatocytes (Figure 4D–F,H). The aforementioned results indicated critical involvement of Sirt1 in the hepatoprotective activity of ERS. 

### 2.5. Knockdown of LKB1 Abrogates ERS-Mediated Activation of AMPK/Nrf2 Pathway

**Figure 5:**
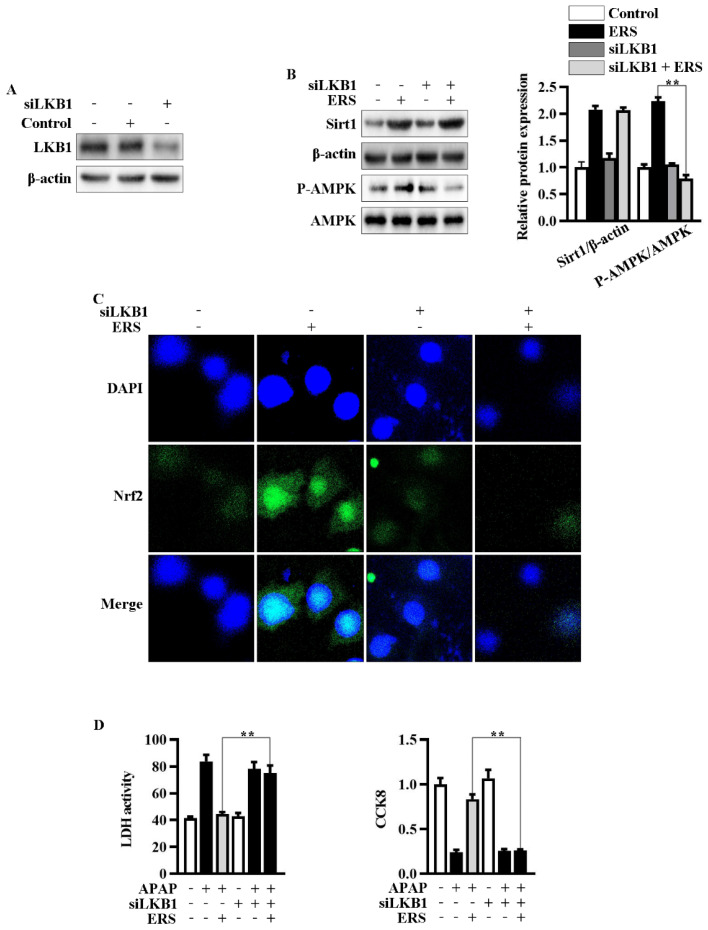
Knockdown of LKB1 abrogated ERS-mediated activation of AMPK/Nrf2 axis. (**A**) Effects of transfecting siLKB1 on levels of LKB1 were evaluated by immunoblotting. (**B**,**C**) AML-12 cells were subjected to the indicated treatments. Then, protein levels of Sirt1 and AMPK phosphorylation were detected by immunoblotting (**B**). Levels of Nrf2 in nuclei were determined by confocal microscope. (**C**). (**D**) Cells were subjected to the indicated treatment. Then, cytotoxic effects were determined by LDH and CCK8 assay. ** *p* < 0.01, compared to the indicated control (cells transfected with si-Con and treated with ERS and APAP).

Then, we explored the roles of enhanced phosphorylation of LKB1 in ERS-mediated potentiation of AMPK/Nrf2. We transfected siLKB1 into AML-12 cells and found that LKB1 expression was effectively knocked down by siLKB1 (Figure 5A). Knockdown of LKB1 abolished ERS-induced AMPK phosphorylation and subsequent Nrf2 nuclear translocation, while upregulation of Sirt1 remained almost unchanged (Figure 5B,C). Additionally, the protective effects of ERS on APAP-caused cytotoxicity in AML-12 cells were also eliminated after transfecting siLKB1 (Figure 5D). These results collectively indicated that ERS activates AMPK/Nrf2 antioxidant axis via promoting LKB1 phosphorylation. Upregulation of Sirt1 expression appears to be a key upstream event resulting in LKB1 phosphorylation. 

### 2.6. ERS Alleviates APAP-Caused Liver Injury in Mice

**Figure 6:**
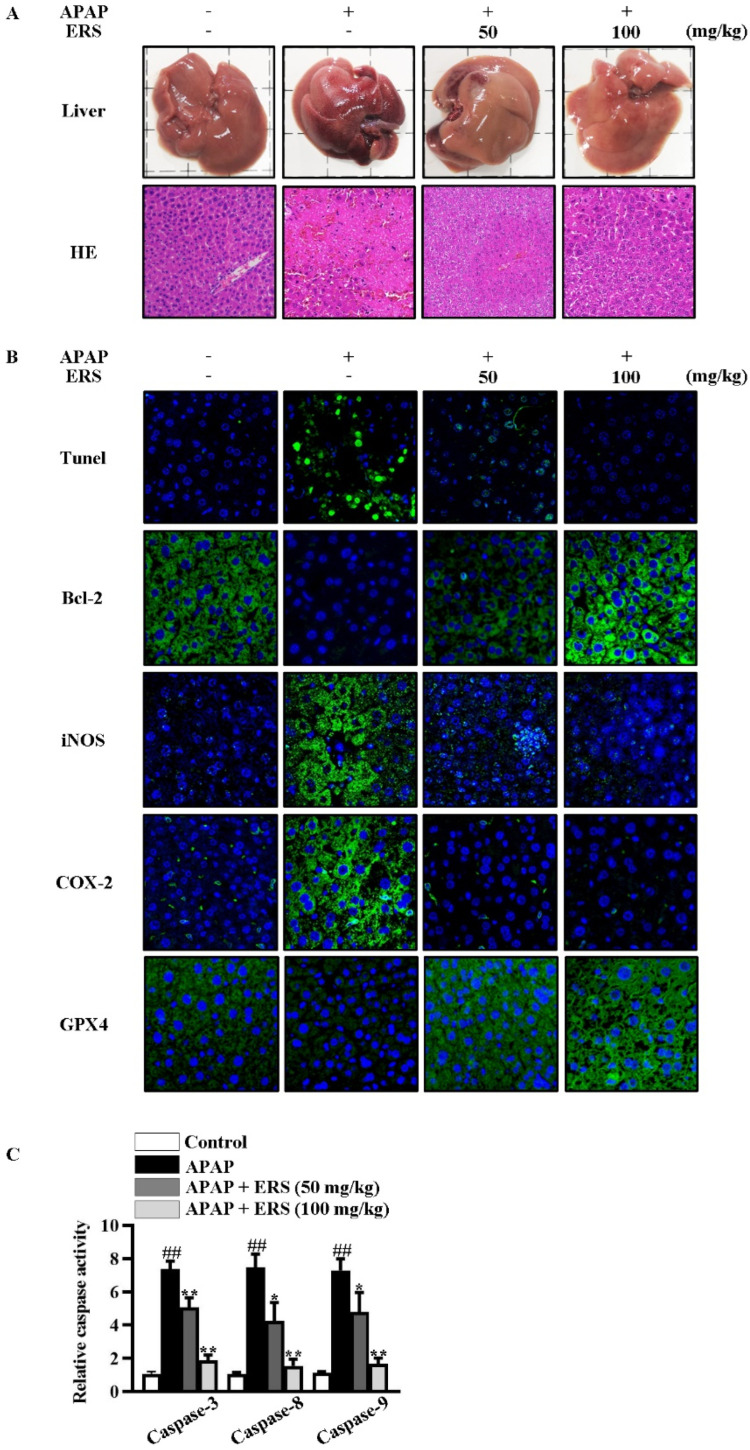
ERS reduced APAP-provoked liver injury in mice. (**A**–**C**) Mice were treated as indicated. Appearances of livers of mice were shown, and liver sections were prepared and subjected to HE staining (**A**). Apoptotic cells in livers were visualized by TUNEL. Hepatic levels of Bcl-2, iNOS, COX-2, and GPX4 were determined by immunohistochemistry staining (**B**). Activities of caspase-3, -8, and -9 in livers were also quantified (**C**). ^##^: *p* < 0.01, compared to the normal mice. * *p* < 0.05, ** *p* < 0.01, compared with the mice receiving APAP alone.

As detected by liver appearance, HE staining, liver index, and serum liver function parameters, APAP-provoked liver injury was ameliorated by ERS (Figure 6A and Table 1). Under APAP stimulation, hepatic levels of inflammatory factors were increased dramatically. Likewise, hepatic levels of inflammation-related factors, such as iNOS and COX-2, were significantly upregulated (Figure 6B). ERS significantly attenuated APAP-provoked intrahepatic inflammation responses (Figure 6B and Table 2). The findings above collectively unveiled that liver injury resulting from APAP intoxication can be improved by ERS.

### 2.7. ERS Reduces Hepatocyte Apoptosis and Ferroptosis in APAP-Intoxicated Mice

As shown by TUNEL staining and caspase activity assay, ERS obviously relieved APAP-induced apoptosis in mouse livers (Figure 6B,C). Consistently, APAP reduced the hepatic expression of anti-apoptotic factor Bcl-2 in mouse livers, which was restored by ERS (Figure 6B). Moreover, levels of ferroptosis indicators were dramatically increased by APAP and declined in response to ERS (Table 3). GPX4 is an inhibitor of ferroptosis. APAP reduced intrahepatic GPX4, and this process was rescued by ERS (Figure 6B). These results indicated that ERS exerts suppressive effects on APAP-induced intrahepatic ferroptosis. 

### 2.8. ERS Modulates Sirt1/LKB1/AMPK/Nrf2 Cascade in Mouse Livers

**Figure 7:**
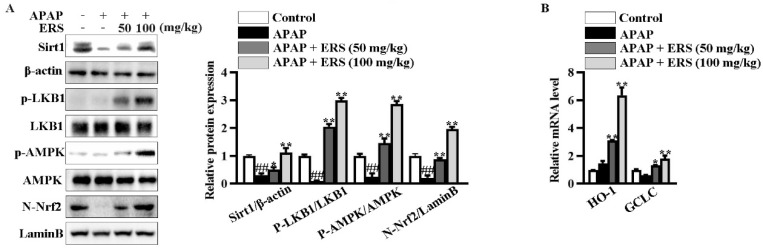
ERS modulated Sirt1/LKB1/AMPK/Nrf2 cascade in mouse livers. (**A**,**B**) Mice were treated as indicated. Hepatic levels of Sirt1 and phosphorylated LKB1 and AMPK in total lysates and Nrf2 in nuclei were detected by immunoblotting (**A**). Transcriptions of HO-1 and GCLC were detected by real-time PCR (**B**). ^##^: *p* < 0.01, compared to the normal group. * *p* < 0.05, ** *p <* 0.01, compared to the mice receiving APAP alone.

In livers of mice receiving APAP, ERS considerably boosted Sirt1 expression, promoted phosphorylations of LKB1 and AMPK, and induced nuclear translocation of Nrf2 (Figure 7A). Consistently, transcriptions of Nrf2 downstream genes, such as HO-1 and GCLC, were upregulated by ERS (Figure 7B). Additionally, as shown in (Table 4), APAP reduced contents of antioxidant factors in mouse livers. This lesion could be relieved by ERS. These observations indicated that the Sirt1/LKB1/AMPK/Nrf2 antioxidant pathway can be activated by ERS in vivo. 

## 3. Discussion

In this study, dosages of ERS were selected based on previous kinds of literature and our pilot experiments [38,39]. The concentration of APAP to induce cytotoxic effects in AML-12 hepatocytes and the dosage of APAP to provoke hepatotoxicity in mice were similar to previous reports [43,44]. We revealed that ERS pretreatment obviously hampered APAP-induced cytotoxicity in AML-12 hepatocytes. The alleviative effects of ERS were comparable to those of NAC, which is a positive control drug [45]. Our further animal experiments showed that ERS relieved APAP-provoked hepatotoxicity in vivo. In this study, after ERS treatment, serum ALT decreased to 27.6% of the APAP-intoxicated mice and AST decreased to 37.3% of the APAP-intoxicated mice. In some of the literature, the same dosage of APAP was used to induce hepatotoxicity, and the alleviative effects of NAC in their report were comparable to those of ERS here, based on the ratio of changes in serum ALT and AST values [46,47]. It is thus reasonable to speculate that the improvement effect of ESR on APAP-induced liver injury in mice is close to that of NAC. Intrahepatic inflammation plays a critical role in APAP-mediated hepatotoxicity. In response to ERS, levels of inflammatory factors and mediators in the livers of mice exposed to APAP were dramatically downregulated. Hepatic expressions of iNOS and COX-2 which promote the synthesis of inflammatory mediators were also decreased. Consistent with our findings, silymarin, an approved hepatoprotective drug, ameliorates APAP-provoked hepatic inflammation and subsequent liver injury [48]. The yield of ERS from dried rose buds and the contents of five representative compounds in ERS were similar to data published previously, suggesting the process of ERS preparation is reliable and stable [49,50]. Our results collectively suggested that ERS has the potential to alleviate APAP liver injury and support the application of ERS as a promising hepatoprotective reagent. Our further studies would systematically analyze the compounds in ERS to clarify its HPLC fingerprint and quality control standards. To widely validate the hepatoprotective activity of ERS, we were going to use normal hepatocytes, such as primary hepatocytes, or different experimental animals, such as C57 mice or SD rats, to assess the ameliorative effects of ERS on APAP hepatotoxicity.

Hepatic oxidative stress is a key factor promoting APAP-provoked liver injury [51]. We found that, under APAP exposure, obvious oxidative stress occurred in both AML-12 hepatocytes and mouse livers, which were dramatically impinged by ERS. Oxidative stress results in cell apoptosis and ferroptosis [52]. Curcumin and cinnamon oil improve APAP-induced hepatotoxicity by suppressing hepatocyte apoptosis [53,54]. Attenuation of hepatocyte ferroptosis is critically involved in kaempferol or ulinastatin-mediated protection against APAP [17,55]. ERS effectively reduced hepatocyte apoptosis and ferroptosis in vitro and in mice, indicating important roles of inhibition of hepatocyte apoptosis and ferroptosis in ERS-mediated hepatoprotection. ERS promoted nuclear translocation of Nrf2 and transcriptions of its responsive genes in hepatocytes, suggesting that ERS activates Nrf2 antioxidative signaling. Similarly, other natural bioactive substances also improve APAP-provoked liver injury via activating Nrf2 [22,56]. It has been unveiled previously that rose flowers enhance intracellular antioxidant capacity [41,57], but little is known about the underlying molecular mechanism. Our findings suggested that potentiating the Nrf2 cascade can be an indispensable contributor to the antioxidant activity of roses. Ellagic acid is a characteristic chemical component in ERS. It positively modulates the Nrf2 cascade in HepG2 hepatoma cells at concentrations of 15 and 30 μM [58]. In our study, ERS (10 and 30 μg/mL) potentiated Nrf2 in AML-12 hepatocytes. Concentrations of ellagic acid in ERS were 2.03 and 6.09 μM. These discrepancies indicated that the hepatoprotective effects of ERS are not equivalent to those of ellagic acid.

Many bioactive substances activate Nrf2 through AMPK [26]. Similar to this literature, ERS boosted AMPK phosphorylation and enhanced its catalytic activity in both AML-12 hepatocytes and the livers of APAP-intoxicated mice. LKB1 imposes positive regulatory effects on AMPK. Accompanied by palmitic acid-induced triglyceride deposition in hepatocytes, obvious downregulation of LKB1 and AMPK phosphorylation can be detected [59]. The activity of the LKB1/AMPK cascade is also reduced during acute liver injury provoked by amino galactose [60]. The data above indicated a possibility that declined hepatic LKB1/AMPK signaling is tightly associated with liver lesions. We found that ERS upregulated LKB1 phosphorylation. Moreover, ERS could not activate the AMPK/Nrf2 axis in hepatocytes or protect them from APAP if expression of LKB1 is knocked down. Our findings above suggest that the potentiation of LKB1 can be a critical contributor to ERS-mediated activation of AMPK/Nrf2. Similarly, compounds such as ganodermandiol, berbamine, and rifampicin activate hepatic AMPK/Nrf2 via upregulating LKB1 phosphorylation [28,61], thereby exerting their hepatoprotective activity. The results above indicated that LKB1 can be a promising target for screening bioactive substances with hepatoprotective potential.

Sirt1 is involved in regulating a series of important biological processes such as oxidative stress, inflammatory response, and cell death [62]. The regulatory effects of Sirt1 on LKB1/AMPK/Nrf2 signaling are receiving more and more attention. As a catalytic activity of Sirt1 is enhanced, phosphorylation of LKB1 and AMPK is thereby considerably upregulated [63]. Additionally, overexpression of Sirt1 in the liver activates the hepatic LKB1/AMPK axis and promotes Nrf2-dependent gene transcription [64,65]. Under APAP stimulation, levels of Sirt1 in the liver decline, which can be further supported by our current study [46,66]. We also unveiled that ERS upregulated Sirt1 expression in the livers of APAP-intoxicated mice. Previous studies unveiled that, in HepG2 hepatoma cells, roses eliminate ethanol-induced Sirt1 downregulation and AMPK dephosphorylation [38]. However, based on their data, it is still uncertain whether roses directly increase Sirt1 or indirectly affect Sirt1 expression levels by reducing ethanol toxicity. We found that ERS significantly boosted Sirt1 expression in AML-12 hepatocytes. Moreover, EX527 and NAM which are two structurally unrelated inhibitors of Sirt1 both blocked ERS-mediated activation of the LKB1/AMPK/Nrf2 axis and amelioration of APAP-induced hepatotoxicity. Additionally, even if an expression of LKB1 was knocked down, ERS still boosted Sirt1 expression in AML-12 cells. This phenomenon suggested that the upregulation of Sirt1 is the upstream event of elevated LKB1 phosphorylation triggered by ERS. This point can be supported by a series of previous studies unveiling that Sirt1 is an upstream regulator of LKB1 activity [63,67,68]. Our findings above collectively indicated that ERS induces LKB1 phosphorylation and activates its downstream AMPK/Nrf2 pathway via upregulating Sirt1. Consistent with our research, many bioactive substances activate Nrf2 cascade through Sirt1 [63,69]. The aforementioned observations suggest that enhancing the hepatic Sirt1/LKB1 cascade appears to be a feasible method for controlling liver diseases. Here, we used a Sirt1 inhibitor in our in vitro experiments. Previous studies showed that Sirt1 inhibitor NAM improved APAP-induced liver injury in mice [70,71]. Therefore, it would be difficult to obtain our expected results, that the inhibition of Sirt1 by its inhibitor eliminated ERS-mediated protection against APAP-induced liver injury in vivo. To confirm the molecular mechanisms underlying the hepatoprotective activity of ERS in vivo, we would try to knockdown/overexpress Sirt1 or LKB1 in hepatocytes of living mice and explore the effects of these alterations on ERS-mediated protection against APAP hepatotoxicity.

## 4. Materials and Methods

### 4.1. Materials

APAP was procured from Aladdin (Shanghai, China). *N*-Acetyl-L-cysteine (NAC), EX527, and NAM from Beyotime Biotechnology (Shanghai, China). The manufacturers of kits and antibodies are provided in the Supplementary Materials.

### 4.2. Preparation of ERS

ERS was prepared and analyzed as described previously [48]. Briefly, 10 g of dry flower buds of *Rosa rugosa* were grounded into powders and extracted in 200 mL of solvent (ethanol/water, 85:15, *v*/*v*) under ultrasound for 30 min at 45 °C. Extract supernatant was collected, filtered, and lyophilized, yielding 1.29 g of ERS. Total polyphenol content in ERS was measured by the corresponding assay kit. The chemical profile of ERS was identified based on previous literature [41] and was quantified by HPLC. Detailed HPLC conditions are provided in the Supplementary Materials.

### 4.3. Cells and Transfection

Mouse AML-12 hepatocytes were cultured and transfected as described previously [72]. Sequences of siRNA targeting mouse LKB1 (si-LKB1) and the corresponding control siRNA (si-Con) were the same as described previously [73]. 

### 4.4. Cytotoxicity Detection and ROS Quantification 

Cytotoxic effects were determined by LDH, CCK8, and MTT assays using the corresponding kit following the manufacturer’s instructions. Intracellular ROS was quantified using the DCFH-DA ROS assay kit. 

### 4.5. Detection of Apoptosis and Ferroptosis

Cell apoptosis was determined by caspase activity assay and flow cytometry analysis using the corresponding kit. Cell ferroptosis was determined by measuring levels of iron, MDA, 4-HNE, and LPO using the corresponding kit.

### 4.6. Immunoblotting, Immunohistochemistry and Immunofluorescence

Immunoblotting, immunohistochemistry, and immunofluorescence were performed as described previously [74].

### 4.7. Quantification of Gene Transcription

Transcriptions of indicated genes were determined by reverse transcription-quantitative PCR. The experimental procedure and primer sets were the same as described previously [73]. 

### 4.8. Animal Experiments

Male Kunming mice (6 weeks old, approximately 25 g) were bought from the Hubei Experimental Animal Research Center (Wuhan, China) and maintained in a standard specific pathogen-free environment. All of our animal experiments were performed under the supervision of the Ethical Committee of Experimental Animal Care at South-Central Minzu University (2020-SCUEC-AEC-014). 

Previous literature reported that the dosages of ESR for mice ranged from 80 to 150 mg/kg [36,75]. Our preliminary research showed that, at a dosage of 100 mg/kg, ERS exerted a convincing ameliorative effect on liver injury induced by APAP. Therefore, the higher dosage of ERS in vivo here was set to 100 mg/kg.

Mice were randomly divided into four groups with eight mice in each group, i.e., a normal group, an APAP model group, a model + ERS (50 mg/kg) group, and a model + ERS (100 mg/kg) group. ERS was administrated via gavage once daily for 7 consecutive days. Two hours after the final ERS gavage, mice in the APAP model group and model + ERS groups were intraperitoneally injected with APAP (300 mg/kg). All mice were sacrificed 24 h later after collecting blood under ether anesthesia. Their livers were immediately collected, properly preserved, and subjected to further experiments.

### 4.9. Statistical Analysis

Data were expressed as mean ± standard deviation (n = 3 unless otherwise indicated). Statistical significance was tested by one-way ANOVA followed by Turkey’s multiple comparisons and considered achieved if *p* < 0.05.

Further descriptions of the materials and methods are provided in the Supplementary Materials.

## 5. Conclusions

**Figure 8:**
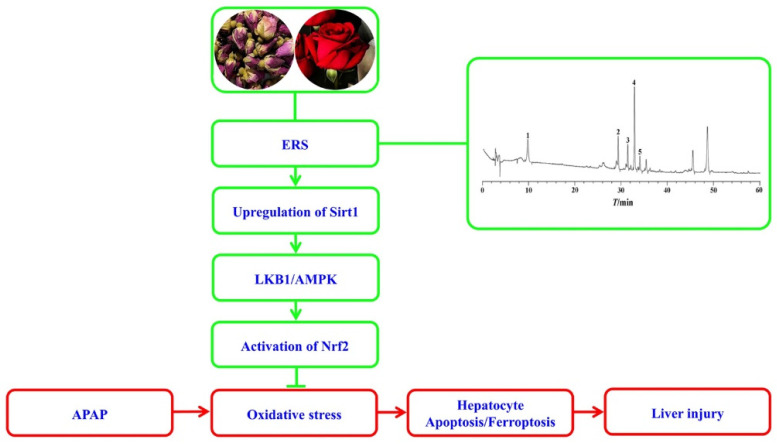
A diagram outlining the proposed molecular cascade contributing to ERS-mediated protection against the hepatotoxicity of APAP.

As summarized in Figure 8, ERS effectively relieves APAP-induced AML-12 hepatocyte demise, including apoptosis and ferroptosis and mouse liver injury. Mechanistically, ERS potentiates the LKB1/AMPK/Nrf2 antioxidant cascade via upregulating intracellular Sirt1 in hepatocytes. 

## Figures and Tables

**Table 1:** Effects of ERS on mouse liver indexes and serum parameters of liver function.

Group	ALT (U·L^−1^)	AST (U·L^−1^)	LDH (U·L^−1^)	Liver Index (%)
Normal	26 ± 5.2	38 ± 6.9	60 ± 10	5.0 ± 0.24
APAP	199 ± 58 ^##^	150 ± 23 ^##^	150 ± 27 ^##^	6.9 ± 0.28 ^##^
APAP + ERS (50 mg/kg)	90 ± 10 **	92 ± 15 *	110 ± 14 **	5.8 ± 0.56 *
APAP + ERS (100 mg/kg)	55 ± 8.7 **	56 ± 12 **	73 ± 14 **	5.2 ± 0.23 **

Data are presented as mean ± SD (n = 8). ^##^ *p* < 0.01, compared with the normal group. * *p* < 0.05, ** *p* < 0.01, compared with the APAP model group. Liver index = Liver weight/whole body weight × 100%.

**Table 2:** Effects of ERS on hepatic levels of inflammatory factors in mice.

Group	TNF-α (pg/mg Prot)	IL-1β (pg/mg Prot)	IL-6 (pg/mg Prot)	PGE2 (pg/mg Prot)	NO (μM/mg Prot)
Normal	155.7 ± 11.6	204.4 ± 17.5	123.5 ± 17.5	231.6 ± 31.3	69.4 ± 12.2
APAP	475.5 ± 24.8 ^##^	307.4 ± 29.4 ^##^	301.5 ± 22.8 ^##^	586.3 ± 75.6 ^##^	142.7 ± 23.7 ^##^
APAP + ERS (50 mg/kg)	250.1 ± 17.1 **	260.4 ± 32.4 **	242.7 ± 10.3 **	386.7 ± 54.3 **	74.5 ± 9.8 **
APAP + ERS (100 mg/kg)	183.7 ± 10.5 **	215.3 ± 36.7 **	141.4 ± 12.4 **	268.2 ± 23.4 **	49.1 ± 8.7 **

Data are presented as mean ± SD (n = 8). ^##^ *p* < 0.01, compared with the normal group. ** *p* < 0.01, compared with the APAP model group.

**Table 3:** Effects of ERS on hepatic levels of ferroptosis parameters in mice.

Group	Iron (μM/μg Tissue)	LPO (nM/mg Prot)	4-HNE (nM/mg Prot)	MDA (nM/mg Prot)
Normal	1.6 ± 0.18	1.8 ± 0.18	1.8 ± 0.54	0.8 ± 0.36
APAP	5.4 ± 0.37 ^##^	2.9 ± 0.16 ^##^	7.8 ± 0.96 ^##^	1.3 ± 0.55 ^##^
APAP + ERS (50 mg/kg)	3.3 ± 0.23 *	2.2 ± 0.12 *	3.7 ± 0.28 **	1.04 ± 0.22 *
APAP + ERS (100 mg/kg)	2.6 ± 0.17 **	2.0 ± 0.11 **	2.7 ± 0.71 **	0.96 ± 0.47 **

Data are presented as mean ± SD (n = 8). ^##^ *p* < 0.01, compared with the normal group. * *p* < 0.05, ** *p* < 0.01, compared with the APAP model group.

**Table 4:** Effects of ERS on hepatic levels of indicators of oxidative stress in mice.

Group	T-AOC (mM/g Prot)	SOD (U/mg Prot)	GSH (μM/g Prot)	GPX-P (μM/mg Prot)	CAT (U/mg Prot)
Normal	1.2 ± 0.15	20 ± 1.4	15 ± 1.54	77 ± 7.2	43 ± 4.3
APAP	0.32 ± 0.09 ^##^	15 ± 1.1 ^##^	6 ± 0.89 ^##^	54 ± 10 ^##^	15 ± 1.8 ^##^
APAP + ERS (50 mg/kg)	0.53 ± 0.18 *	17 ± 1.5 **	10 ± 1.24 *	73 ± 14 **	18 ± 2.1 **
APAP + ERS (100 mg/kg)	0.77 ± 0.19 **	19 ± 1.7 **	13 ± 1.66 **	82 ± 12 **	34 ± 2.5 **

Data are presented as mean ± SD (n = 8). ^##^ *p* < 0.01, compared with the normal group. * *p* < 0.05, ** *p* < 0.01, compared with the APAP model group.

## Data Availability

Data presented here are available from the corresponding author upon reasonable request.

## Supplementary Materials

The following supporting information can be downloaded at: https://www.mdpi.com/article/10.3390/molecules28217307/s1. Reference [76] is cited in the supplementary materials.

## Abbreviations

4-HNE	4-hydroxynonenal
ALT	alanine aminotransferase
AMPK	AMP-activated protein kinase
APAP	acetaminophen
AST	aspartate aminotransferase
Bax	BCL2-associated X protein
Bcl-2	B-cell lymphoma-2
CAT	catalase
COX2	cyclooxygenase-2
ERS	ethanol extract of *Rosa rugosa*
EX527	6-Chloro-2,3,4,9-tetrahydro-1H-Carbazole-1-carboxamide
GCLC	glutamate-cysteine ligase
GPX	glutathione peroxidase
GPX4	glutathione peroxidase 4
GSH	glutathione
HE	hematoxylin-eosin
HO-1	heme oxygenase 1
HPLC	high-performance liquid chromatography
IL-1β	interleukin-1β
IL-6	interleukin-6
iNOS	inducible nitric oxide synthase
Keap1	kelch-like ECH-associated protein 1
LDH	lactate dehydrogenase
LKB1	liver kinase B1
LPO	lipid peroxidation
MDA	malondialdehyde
NAC	N-acetyl cysteine
NAM	nicotinamide
NO	nitric oxide
Nrf2	nuclear factor erythroid 2-related factor 2
*p*-AMPK	phosphorylated AMPK
*p*-LKB1	phosphorylated LKB1
PEG2	prostaglandin E2
ROS	reactive oxygen species
Sirt1	sirtuin 1
SOD	superoxide dismutase
T-AOC	total antioxidant capacity
TNF-α	tumor necrosis factor-α

## References

[1] Ramachandran A., Jaeschke H. (2019). Acetaminophen Hepatotoxicity. Semin. Liver Dis..

[2] Yan M., Huo Y., Yin S., Hu H. (2018). Mechanisms of acetaminophen-induced liver injury and its implications for therapeutic interventions. Redox Biol..

[3] Ghanem C.I., Pérez M.J., Manautou J.E., Mottino A.D. (2016). Acetaminophen from liver to brain: New insights into drug pharmacological action and toxicity. Pharmacol. Res..

[4] Chiew A.L., Gluud C., Brok J., Buckley N.A. (2018). Interventions for paracetamol (acetaminophen) overdose. Cochrane Database Syst. Rev..

[5] Smilkstein M.J., Knapp G.L., Kulig K.W., Rumack B.H. (1988). Efficacy of oral N-acetylcysteine in the treatment of acetaminophen overdose. Analysis of the national multicenter study (1976 to 1985). N. Engl. J. Med..

[6] Jaeschke H., Akakpo J.Y., Umbaugh D.S., Ramachandran A. (2020). Novel therapeutic approaches against acetaminophen-induced liver injury and acute liver failure. Toxicol. Sci..

[7] Guicciardi M.E., Gores G.J. (2005). Apoptosis: A mechanism of acute and chronic liver injury. Gut.

[8] Cao P., Sun J., Sullivan M.A., Huang X., Wang H., Zhang Y., Wang N., Wang K. (2018). Angelica sinensis polysaccharide protects against acetaminophen-induced acute liver injury and cell death by suppressing oxidative stress and hepatic apoptosis in vivo and in vitro. Int. J. Biol. Macromol..

[9] Schattenberg J.M., Galle P.R., Schuchmann M. (2006). Apoptosis in liver disease. Liver Int..

[10] Scaffidi C., Fulda S., Srinivasan A., Friesen C., Li F., Tomaselli K.J., Debatin K.M., Krammer P.H., Peter M.E. (1998). Two CD95 (APO-1/Fas) signaling pathways. EMBO J..

[11] Ursini F., Maiorino M. (2020). Lipid peroxidation and ferroptosis: The role of GSH and GPx4. Free Radic. Bio. Med..

[12] Latunde-Dada G.O. (2017). Ferroptosis: Role of lipid peroxidation, iron and ferritinophagy. BBA-Gen. Subj..

[13] Dang Q., Sun Z., Wang Y., Wang L., Liu Z., Han X. (2022). Ferroptosis: A double-edged sword mediating immune tolerance of cancer. Cell Death Dis..

[14] Niu B., Lei X., Xu Q., Ju Y., Xu D., Mao L., Li J., Zheng Y., Sun N., Zhang X. (2022). Protecting mitochondria via inhibiting VDAC1 oligomerization alleviates ferroptosis in acetaminophen-induced acute liver injury. Cell Biol. Toxicol..

[15] Yamada N., Karasawa T., Kimura H., Watanabe S., Komada T., Kamata R., Sampilvanjil A., Ito J., Nakagawa K., Kuwata H. (2020). Ferroptosis driven by radical oxidation of n-6 polyunsaturated fatty acids mediates acetaminophen-induced acute liver failure. Cell Death Dis..

[16] Lőrincz T., Jemnitz K., Kardon T., Mandl J., Szarka A. (2015). Ferroptosis is Involved in Acetaminophen Induced Cell Death. Pathol. Oncol. Res..

[17] Wang C., Liu T., Tong Y., Cui R., Qu K., Liu C., Zhang J. (2021). Ulinastatin protects against acetaminophen-induced liver injury by alleviating ferroptosis via the SIRT1/NRF2/HO-1 pathway. Am. J. Transl. Res..

[18] Cai X., Hua S., Deng J., Du Z., Zhang D., Liu Z., Khan N.U., Zhou M., Chen Z. (2022). Astaxanthin Activated the Nrf2/HO-1 Pathway to Enhance Autophagy and Inhibit Ferroptosis, Ameliorating Acetaminophen-Induced Liver Injury. ACS Appl. Mater. Interfaces.

[19] Li H., Weng Q., Gong S., Zhang W., Wang J., Huang Y., Li Y., Guo J., Lan T. (2023). Kaempferol prevents acetaminophen-induced liver injury by suppressing hepatocyte ferroptosis via Nrf2 pathway activation. Food Funct..

[20] Peace C.G., O’Neill L.A. (2022). The role of itaconate in host defense and inflammation. J. Clin. Investig..

[21] Vomund S., Schäfer A., Parnham M.J., Brüne B., von Knethen A. (2017). Nrf2, the Master Regulator of Anti-Oxidative Responses. Int. J. Mol. Sci..

[22] Lv H., Hong L., Tian Y., Yin C., Zhu C., Feng H. (2019). Corilagin alleviates acetaminophen-induced hepatotoxicity via enhancing the AMPK/GSK3β-Nrf2 signaling pathway. Cell Commun. Signal..

[23] Liu J., Wu K.C., Lu Y.F., Ekuase E., Klaassen C.D. (2013). Nrf2 protection against liver injury produced by various hepatotoxicants. Oxid. Med. Cell. Longev..

[24] Liang K.J., Woodard K.T., Weaver M.A., Gaylor J.P., Weiss E.R., Samulski R.J. (2017). AAV-Nrf2 Promotes Protection and Recovery in Animal Models of Oxidative Stress. Mol. Ther..

[25] Gao Z., Yi W., Tang J., Sun Y., Huang J., Lan T., Dai X., Xu S., Jin Z.G., Wu X. (2022). Urolithin A protects against acetaminophen-induced liver injury in mice via sustained activation of Nrf2. Int. J. Biol. Sci..

[26] Didamoony M.A., Atwa A.M., Abd El-Haleim E.A., Ahmed L.A. (2022). Bromelain ameliorates D-galactosamine-induced acute liver injury: Role of SIRT1/LKB1/AMPK, GSK3β/Nrf2 and NF-κB p65/TNF-α/caspase-8, -9 signalling pathways. J. Pharm. Pharmacol..

[27] Joo M.S., Kim W.D., Lee K.Y., Kim J.H., Koo J.H., Kim S.G. (2016). AMPK Facilitates Nuclear Accumulation of Nrf2 by Phosphorylating at Serine 550. Mol. Cell. Biol..

[28] Lee E.H., Baek S.Y., Park J.Y., Kim Y.W. (2020). Rifampicin activates AMPK and alleviates oxidative stress in the liver as mediated with Nrf2 signaling. Chem.-Biol. Interact..

[29] BinMowyna M.N., AlFaris N.A. (2021). Kaempferol suppresses acetaminophen-induced liver damage by upregulation/activation of SIRT1. Pharm. Biol..

[30] Tang Y., Zhao R., Pu Q., Jiang S., Yu F., Yang Z., Han T. (2023). Investigation of nephrotoxicity on mice exposed to polystyrene nanoplastics and the potential amelioration effects of DHA-enriched phosphatidylserine. Sci. Total Environ..

[31] Li S.N., Yu Y.L., Wang B.Y., Qiao S.Y., Hu M.M., Wang H., Fu C.N., Dong B. (2022). Overexpression of G Protein-Coupled Receptor 40 Protects Obesity-Induced Cardiomyopathy Through the SIRT1/LKB1/AMPK Pathway. Hum. Gene Ther..

[32] Li M., Wang S., Li X., Kou R., Wang Q., Wang X., Zhao N., Zeng T., Xie K. (2018). Diallyl sulfide treatment protects against acetaminophen-/carbon tetrachloride-induced acute liver injury by inhibiting oxidative stress, inflammation and apoptosis in mice. Toxicol. Res..

[33] Li X., Park S.J., Jin F., Deng Y., Yang J.H., Chang J.H., Kim D.Y., Kim J.A., Lee Y.J., Murakami M. (2018). Tanshinone IIA suppresses FcεRI-mediated mast cell signaling and anaphylaxis by activation of the Sirt1/LKB1/AMPK pathway. Biochem. Pharmacol..

[34] Yu M., Zhou M., Li J., Zong R., Yan Y., Kong L., Zhu Q., Li C. (2022). Notch-activated mesenchymal stromal/stem cells enhance the protective effect against acetaminophen-induced acute liver injury by activating AMPK/SIRT1 pathway. Stem Cell Res. Ther..

[35] Liu X., Zhao H., Luo C., Du D., Huang J., Ming Q., Jin F., Wang D., Huang W. (2019). Acetaminophen Responsive miR-19b Modulates SIRT1/Nrf2 Signaling Pathway in Drug-Induced Hepatotoxicity. Toxicol. Sci..

[36] Ng T.B., Gao W., Li L., Niu S.M., Zhao L., Liu J., Shi L.S., Fu M., Liu F. (2005). Rose (*Rosa rugosa*)-flower extract increases the activities of antioxidant enzymes and their gene expression and reduces lipid peroxidation. Biochem. Cell Biol..

[37] Liu L., Yasen M., Tang D., Ye J., Aisa H.A., Xin X. (2018). Polyphenol-enriched extract of *Rosa rugosa* Thunb regulates lipid metabolism in diabetic rats by activation of AMPK pathway. Biomed. Pharmacother..

[38] Zhou Q., Wang L., Liu B., Xiao J., Cheng K.W., Chen F., Wang M. (2021). Tricoumaroylspermidine from rose exhibits inhibitory activity against ethanol-induced apoptosis in HepG2 cells. Food Funct..

[39] Chen T., Li J., Chen J., Song H., Yang C. (2015). Anti-hyperplasia effects of *Rosa rugosa* polyphenols in rats with hyperplasia of mammary gland. Environ. Toxicol. Pharmacol..

[40] Shu G., Lei X., Lei Y., Zhang T., Sun H., Wang C., Song A., Deng X. (2023). A characterized ethanol extract of *Rosa rugosa* inhibits hepatic stellate cell activation through elevating Hint1 and subsequent upregulation of Smad7. J. Funct. Foods.

[41] Nijat D., Lu C.F., Lu J.J., Abdulla R., Hasan A., Aidarhan N., Aisa H.A. (2021). Spectrum-effect relationship between UPLC fingerprints and antidiabetic and antioxidant activities of *Rosa rugosa*. J. Chromatogr. B.

[42] Wan H., Yu C., Han Y., Guo X., Ahmad S., Tang A., Wang J., Cheng T., Pan H., Zhang Q. (2018). Flavonols and Carotenoids in Yellow Petals of Rose Cultivar (Rosa ‘Sun City’): A Possible Rich Source of Bioactive Compounds. J. Agric. Food Chem..

[43] Shu G., Qiu Y., Hao J., Fu Q., Deng X. (2019). γ-Oryzanol alleviates acetaminophen-induced liver injury: Roles of modulating AMPK/GSK3β/Nrf2 and NF-κB signaling pathways. Food Funct..

[44] Wang Z., Zhao Y., Sun R., Sun Y., Liu D., Lin M., Chen Z., Zhou J., Lv L., Tian X. (2020). circ-CBFB upregulates p66Shc to perturb mitochondrial dynamics in APAP-induced liver injury. Cell Death Dis..

[45] Raghu G., Berk M., Campochiaro P.A., Jaeschke H., Marenzi G., Richeldi L., Wen F.Q., Nicoletti F., Calverley P.M.A. (2021). The Multifaceted Therapeutic Role of N-Acetylcysteine (NAC) in Disorders Characterized by Oxidative Stress. Curr. Neuropharmacol..

[46] Yang R., Song C., Chen J., Zhou L., Jiang X., Cao X., Sun Y., Zhang Q. (2020). Limonin ameliorates acetaminophen-induced hepatotoxicity by activating Nrf2 antioxidative pathway and inhibiting NF-κB inflammatory response via upregulating Sirt1. Phytomedicine.

[47] Xiang J., Wang J., Xie H., Liu Y., Bai Y., Che Q., Cao H., Huang G., Guo J., Su Z. (2021). Protective effect and mechanism of chitooligosaccharides on acetaminophen-induced liver injury. Food Funct..

[48] Papackova Z., Heczkova M., Dankova H., Sticova E., Lodererova A., Bartonova L., Poruba M., Cahova M. (2018). Silymarin prevents acetaminophen-induced hepatotoxicity in mice. PLoS ONE.

[49] Piotrowicz Z., Tabisz Ł., Waligórska M., Pankiewicz R., Łęska B. (2021). Phenol-rich alternatives for Rosa x damascena Mill. Efficient phytochemical profiling using different extraction methods and colorimetric assays. Sci. Rep..

[50] Izcara S., Perestrelo R., Morante-Zarcero S., Câmara J.S., Sierra I. (2022). High throughput analytical approach based on μQuEChERS combined with UHPLC-PDA for analysis of bioactive secondary metabolites in edible flowers. Food Chem..

[51] Du K., Ramachandran A., Jaeschke H. (2016). Oxidative stress during acetaminophen hepatotoxicity: Sources, pathophysiological role and therapeutic potential. Redox Biol..

[52] Yu Y., Yan Y., Niu F., Wang Y., Chen X., Su G., Liu Y., Zhao X., Qian L., Liu P. (2021). Ferroptosis: A cell death connecting oxidative stress, inflammation and cardiovascular diseases. Cell Death Discov..

[53] Li G., Chen J.B., Wang C., Xu Z., Nie H., Qin X.Y., Chen X.M., Gong Q. (2013). Curcumin protects against acetaminophen-induced apoptosis in hepatic injury. World J. Gastroenterol..

[54] Hussain S., Ashafaq M., Alshahrani S., Siddiqui R., Ahmed R.A., Khuwaja G., Islam F. (2020). Cinnamon oil against acetaminophen-induced acute liver toxicity by attenuating inflammation, oxidative stress and apoptosis. Toxicol. Rep..

[55] Su Y., Zhao B., Zhou L., Zhang Z., Shen Y., Lv H., AlQudsy L.H.H., Shang P. (2020). Ferroptosis, a novel pharmacological mechanism of anti-cancer drugs. Cancer Lett..

[56] Etemadi Y., Akakpo J.Y., Ramachandran A., Jaeschke H. (2023). Nrf2 as a therapeutic target in acetaminophen hepatotoxicity: A case study with sulforaphane. J. Biochem. Mol. Toxicol..

[57] Dina E., Sklirou A.D., Chatzigeorgiou S., Manola M.S., Cheilari A., Louka X.P., Argyropoulou A., Xynos N., Skaltsounis A.L., Aligiannis N. (2021). An enriched polyphenolic extract obtained from the by-product of Rosa damascena hydrodistillation activates antioxidant and proteostatic modules. Phytomedicine.

[58] Ding X., Jian T., Wu Y., Zuo Y., Li J., Lv H., Ma L., Ren B., Zhao L., Li W. (2019). Ellagic acid ameliorates oxidative stress and insulin resistance in high glucose-treated HepG2 cells via miR-223/keap1-Nrf2 pathway. Biomed. Pharmacother..

[59] Liang Y., Zhang Z., Tu J., Wang Z., Gao X., Deng K., El-Samahy M.A., You P., Fan Y., Wang F. (2021). γ-Linolenic Acid Prevents Lipid Metabolism Disorder in Palmitic Acid-Treated Alpha Mouse Liver-12 Cells by Balancing Autophagy and Apoptosis via the LKB1-AMPK-mTOR Pathway. J. Agric. Food Chem..

[60] Maillet V., Boussetta N., Leclerc J., Fauveau V., Foretz M., Viollet B., Couty J.P., Celton-Morizur S., Perret C., Desdouets C. (2018). LKB1 as a Gatekeeper of Hepatocyte Proliferation and Genomic Integrity during Liver Regeneration. Cell Rep..

[61] Li B., Lee D.S., Kang Y., Yao N.Q., An R.B., Kim Y.C. (2013). Protective effect of ganodermanondiol isolated from the Lingzhi mushroom against tert-butyl hydroperoxide-induced hepatotoxicity through Nrf2-mediated antioxidant enzymes. Food Chem. Toxicol..

[62] Choi S.H., Kim Y.W., Kim S.G. (2010). AMPK-mediated GSK3beta inhibition by isoliquiritigenin contributes to protecting mitochondria against iron-catalyzed oxidative stress. Biochem. Pharmacol..

[63] Hou X., Xu S., Maitland-Toolan K.A., Sato K., Jiang B., Ido Y., Lan F., Walsh K., Wierzbicki M., Verbeuren T.J. (2008). SIRT1 regulates hepatocyte lipid metabolism through activating AMP-activated protein kinase. J. Biol. Chem..

[64] Gautam S., Zhang L., Lee C., Arnaoutova I., Chen H.D., Resaz R., Eva A., Mansfield B.C., Chou J.Y. (2023). Molecular mechanism underlying impaired hepatic autophagy in glycogen storage disease type Ib. Hum. Mol. Genet..

[65] Yan T., Huang J., Nisar M.F., Wan C., Huang W. (2019). The Beneficial Roles of SIRT1 in Drug-Induced Liver Injury. Oxid. Med. Cell. Longev..

[66] Rada P., Pardo V., Mobasher M.A., García-Martínez I., Ruiz L., González-Rodríguez Á., Sanchez-Ramos C., Muntané J., Alemany S., James L.P. (2018). SIRT1 Controls Acetaminophen Hepatotoxicity by Modulating Inflammation and Oxidative Stress. Antioxid. Redox Signal..

[67] Yang Y., Li W., Liu Y., Sun Y., Li Y., Yao Q., Li J., Zhang Q., Gao Y., Gao L. (2014). Alpha-lipoic acid improves high-fat diet-induced hepatic steatosis by modulating the transcription factors SREBP-1, FoxO1 and Nrf2 via the SIRT1/LKB1/AMPK pathway. J. Nutr. Biochem..

[68] Price N.L., Gomes A.P., Ling A.J., Duarte F.V., Martin-Montalvo A., North B.J., Agarwal B., Ye L., Ramadori G., Teodoro J.S. (2012). SIRT1 is required for AMPK activation and the beneficial effects of resveratrol on mitochondrial function. Cell Metab..

[69] Li D., Cui Y., Wang X., Liu F., Li X. (2021). Apple polyphenol extract alleviates lipid accumulation in free-fatty-acid-exposed HepG2 cells via activating autophagy mediated by SIRT1/AMPK signaling. Phytother. Res..

[70] Shi Y., Zhang L., Jiang R., Chen W., Zheng W., Chen L., Tang L., Li L., Li L., Tang W. (2012). Protective effects of nicotinamide against acetaminophen-induced acute liver injury. Int. Immunopharmacol..

[71] Xu J., Zhang L., Jiang R., Hu K., Hu D., Liao C., Jiang S., Yang Y., Huang J., Tang L. (2021). Nicotinamide improves NAD+ levels to protect against acetaminophen-induced acute liver injury in mice. Hum. Exp. Toxicol..

[72] Guo H., Sun J., Li D., Hu Y., Yu X., Hua H., Jing X., Chen F., Jia Z., Xu J. (2019). Shikonin attenuates acetaminophen-induced acute liver injury via inhibition of oxidative stress and inflammation. Biomed. Pharmacother..

[73] Shu G., Sun H., Zhang T., Zhu A., Lei X., Wang C., Song A., Deng X. (2023). Theaflavine inhibits hepatic stellate cell activation by modulating the PKA/LKB1/AMPK/GSK3β cascade and subsequently enhancing Nrf2 signaling. Eur. J. Pharmacol..

[74] Hwang S.L., Li X., Lu Y., Jin Y., Jeong Y.T., Kim Y.D., Lee I.K., Taketomi Y., Sato H., Cho Y.S. (2013). AMP-activated protein kinase negatively regulates FcεRI-mediated mast cell signaling and anaphylaxis in mice. J. Allergy Clin. Immunol..

[75] Baiyisaiti A., Wang Y., Zhang X., Chen W., Qi R. (2019). *Rosa rugosa* flavonoids exhibited PPARα agonist-like effects on genetic severe hypertriglyceridemia of mice. J. Ethnopharmacol..

[76] Zhong L., Gustavsson K.E., Oredsson S., Głąb B., Yilmaz J.L., Olsson M.E. (2016). Determination of free and esterified carotenoid composition in rose hip fruit by HPLC-DAD-APCI(+)-MS. Food Chem..

