# Supplementary material for: Ethanol Extract of Rosa rugosa Ameliorates Acetaminophen-Induced Liver Injury via Upregulating Sirt1 and Subsequent Potentiation of LKB1/AMPK/Nrf2 Cascade in Hepatocytes
Source: Molecules. 2023 Oct 28;28(21):7307. doi: 10.3390/molecules28217307 (PMC10649261; doi:10.3390/molecules28217307)
Supplement: Supplementary file 1 [file molecules-28-07307-s001.zip › Supplementary materials.pdf]

Supplementary materials and methods

1. Kits and antibodies

Biochemical assay kits for alanine aminotransferase (ALT), aspartate aminotransferase (AST), lactate dehydrogenase (LDH), superoxide dismutase (SOD), malondialdehyde (MDA), lipid hydroperoxide (LPO), total antioxidant capacity (T-AOC), glutathione peroxidase (GSH-PX), glutathione (GSH) and MTT cell proliferation and cytotoxicity (MTT) caspase-3 activity, caspase-8 activity and caspase-9 activity were purchased from Nanjing Jian Cheng Bioengineering Institute (Nanjing, China). N-Acetyl-L-cysteine (NAC), EX527, CCK-8, NAM, DCFH-DA, TUNEL apoptosis assay kit, reactive oxygen species (ROS) assay kit, DAPI staining solution, and rabbit antibodies for Bcl-2, iNOS, GPX4 were obtained from Beyotime Biotechnology (Shanghai, China). Enzyme-linked immunosorbent assay (ELISA) kits for mouse tumor necrosis factor-alpha (TNF-α), interleukin-1β (IL-1β), interleukin-6 (IL-6), prostaglandin E2 (PGE2), and 4-hydroxynonenal (4-HNE) were purchased from Jianglai Biotechnology (Wuhan, Hubei, China). Biochemical assay kits for intracellular iron colorimetric (Iron) and reactive oxygen species (DCFH-DA) were obtained from Applygen (Beijing, China)

Primary antibodies against Sirt1, p-LKB1, AMPK, p-AMPK and Nrf2 were bought from Abclonal (Wuhan, Hubei, China). Primary antibodies against LKB1, Lamin B and β-actin were purchased from Proteintech (Wuhan, Hubei, China). Secondary antibodies including goat anti-rabbit HRP-conjugated IgG and goat anti-mouse HRP-conjugated IgG were obtained from Abclonal (Wuhan, Hubei, China).

2. Analysis of ERS by HPLC

Samples were analyzed by HPLC as described previously with slight modifications [1,2].

A C18 Zorbax SB column (150 mm × 4.6 mm, 5 μm; Agilent Technologies) was kept at 25 °C. The testing wavelength was adjusted to 254 nm. The mobile phases were acetonitrile (A) and 0.1% phosphoric acid aqueous solution (B) at a flow-rate of 1 mL/min. The procedure of the gradient elution was 0% -5% A, 0 ~ 10 min; 5% -30% A, 10 ~ 40 min; 30% A, 40 ~ 50 min; and 100% A, 50 ~ 60 min. The injection volume was 10 μL and the procedure was repeated three times for each sample.

3. Histopathological examinations

Mouse livers were immediately fixed in 4% paraformaldehyde, cut into sections (5 μm), and subjected to HE staining and TUNEL using the corresponding kit.

4. Biochemical parameter determination and ELISA

Activities of ALT, AST, and LDH in serum and levels of T-AOC, SOD, GSH, GPX, CAT, and NO were determined by the corresponding biochemical assay kit. Levels of TNF-α, IL-6, IL-1β, and PGE2 in livers were measured using the corresponding ELISA kit.

References

1. Nijat, D.; Lu, C.F.; Lu, J.J.; Abdulla, R.; Hasan, A.; Aidarhan, N.; Aisa, H.A.. Spectrum-effect relationship between UPLC fingerprints and antidiabetic and antioxidant activities of Rosa rugosa. *J. Chromatogr. B. Analyt. Technol. Biomed. Life Sci.* **2021,** *1179*, 122843.

2. Zhong, L.; Gustavsson, K.E.; Oredsson, S.; Głąb, B.; Yilmaz J.L.; Olsson, M.E. Determination of free and esterified carotenoid composition in rose hip fruit by HPLC-DAD-APCI(+)-MS. *Food Chem.* **2016**, *210,* 541-550.
